# Comparison of MicroRNA Profiles in Extracellular Vesicles from Small and Large Goat Follicular Fluid

**DOI:** 10.3390/ani11113190

**Published:** 2021-11-08

**Authors:** Qiang Ding, Miaohan Jin, Peter Kalds, Chunhua Meng, Huili Wang, Jifeng Zhong, Xiaolong Wang, Yulin Chen

**Affiliations:** 1 Institute of Animal Science, Jiangsu Academy of Agricultural Sciences, Nanjing 210014, China; dingqiang198907@163.com (Q.D.); mengchunhua@jaas.com.cn (C.M.); wanghuili318@163.com (H.W.); zhongjifeng64@sina.cn (J.Z.); 2 Key Laboratory of Animal Genetics, Breeding and Reproduction of Shaanxi Province, College of Animal Science and Technology, Northwest A&F University, Xianyang 712100, China; jinmiaohan@nwafu.edu.cn (M.J.); peterkalds@nwafu.edu.cn (P.K.); 3 Department of Animal and Poultry Production, Faculty of Environmental Agricultural Sciences, Arish University, El-Arish 45511, Egypt

**Keywords:** extracellular vesicles, follicular fluid, small RNA sequence, miRNAs

## Abstract

**Simple Summary:**

Ovarian follicular development is associated with ovulation and is further related to litter size in goats. Extracellular vesicles (EVs) derived from miRNAs within follicular fluid undergo dynamic changes, and, together with follicle growth, may be considered as potential regulators of follicular development. However, the function and changes in EVs remain ambiguous. Here, we identified miRNA changes in EVs from small to large goat follicular fluid. Using bioinformatics tools, we demonstrated the existence of differentially expressed miRNAs in EVs from follicles of different sizes that are responsible for an altered biological effect. This study contributes to a better understanding of follicular development in goats.

**Abstract:**

Extracellular vesicles (EVs), which exist in the follicular fluid of ruminant ovaries, are considered as cargo carriers for the transfer of biomolecules to recipient cells. However, the functions and changes in EVs in antral follicles remain ambiguous. In the present study, we isolated and characterized EVs from goat follicular fluid by means of differential ultracentrifugation and Western blotting of marker proteins. Bioinformatics tools were used to detect miRNA expression levels in EVs. Different miRNA expression patterns of EVs exist in small to large follicles. Thirteen differentially expressed miRNAs (seven upregulated and six downregulated) were identified and used for analysis. A total of 1948 predicted target genes of 13 miRNAs were mapped to signaling pathways, and three significantly enriched pathways (FoxO, MAPK, and PI3K-AKT signaling pathways) were involved in follicular development, as revealed by KEGG enrichment analysis. Our findings suggest that EVs in follicular fluid play biofunctional roles during follicular development in goats.

## 1. Introduction

Multiple biological processes are involved in follicular development, including complex signaling pathways and bidirectional communication between somatic cells and oocytes [1]. Large numbers of follicles are produced in mammalian ovaries; however, only a few follicles are selected to ovulate. Typically, the follicles are divided into several stages, including the preantral and antral follicles. Large antral follicles tended to ovulate. Follicular fluid (FF), a product of blood plasma that crosses the ‘blood–follicle barrier’, fills the antral follicles and also contains other molecular components secreted from follicular cells [2]. The content of FF is often considered an indicator of oocyte quality and competence, containing proteins, hormones, nucleic acids (DNAs and RNAs), and other biomolecules similar to plasma [3]. Additionally, FF provides a perfect microenvironment for cell-to-cell communication between somatic cells and oocytes. Therefore, it is necessary to examine the content changes during follicular development.

Extracellular vesicles (EVs) are present in all bodily fluids, including ovarian FF [4,5], and have been recognized as indicators of cell communication [6]. Based on their diameter, EVs are divided into two types: exosomes (~30–150 nm) and microvesicles (~100–1000 nm). In addition, EVs are often identified by protein biomarkers, such as CD 63 and TSG 101, and their morphology [7]. Recently, EVs have also been considered as cargo vessels to transfer biomolecules to recipient cells, such as DNA, RNAs (mRNA, small RNAs (sRNA), and long non-coding RNA (lncRNA), and proteins. Recent evidence has verified that EVs might support oocyte maturation [8], embryo implantation, follicular development, and other roles in reproductive processes [9]. A previous study in bovines reported that EVs from FF (FF-EVs) of small follicles significantly induced cumulus expansion compared to those from large follicles [10]. However, in some other animals, such as goats, the biofunctions of FF-EVs in reproduction are still unexplored. 

EVs are often considered as natural carriers of non-coding RNAs, including microRNAs (miRNAs), which can transport their RNA content to neighboring cells or over a long distance to recipient cells by blood circulation [11]. The miRNAs are a class of approximately 22 nt non-coding RNAs that bind to the mRNA sequence of target genes to affect protein coding that regulates biological functions. There are different types and expression levels of miRNAs in ruminant follicles during follicular development. The miRNA content in EVs is distinct in bovine follicles exposed to different estrous cycle stages [8]. Several studies have shown that extracellular miRNAs in FF from follicles of different sizes exhibit different expression profiles [7,12]. Moreover, the differentially expressed miRNAs are predicted to target genes that regulate the pathways associated with follicular development, meiotic resumption, and ovulation [13]. Therefore, we hypothesized that the expression of miRNA in EVs changes along with follicle growth and may play critical roles in the regulation of ovarian functions via miRNA transfer. 

Cell-to-cell communication during ovarian follicular development involves complex molecular signaling pathways. EVs participate in these molecular processes through their secretion and uptake by follicular cells. To understand the role and importance of EVs during follicle growth in goat ovaries better, we endeavored to identify the EVs and clarify the miRNA changes in FF of small to large follicles. The findings of this study provide new insights into the content changes of goat FF and the molecular mechanisms of follicular development.

## 2. Materials and Methods

### 2.1. FF Collection and EVs Isolation

Goat ovaries were collected from Hengshan slaughterhouse (Shaanxi Province, China), which has a specialty for Shaanbei white cashmere goat in China, and transported to the laboratory in 0.9% physiological saline solution at approximately 25 °C. Ovaries with obvious follicles at the surface were selected for the isolation of FF and GCs. Based on diameter, follicles were divided into two groups: small follicles (<3 mm) and large follicles (>5 mm) (Figure S1). FF was collected in a 1 mL syringe from small or large follicles, and then mixed with phosphate-buffered saline (PBS) in a 1:1 ratio. EVs were isolated using the differential ultracentrifugation method. Before ultracentrifugation, all diluted FF was spun at 300× *g* for 10 min to separate the cells (oocytes and GCs). Second, the supernatant FF was centrifuged at 2000× *g* for 20 min to remove the remaining dead cells and then centrifuged at 12,000× *g* for 45 min to precipitate the cellular debris and large particles, and immediately followed by filtration through a 0.22 μm filter to further remove large vesicles with diameters larger than 220 nm. Ultracentrifugation was used to isolate extracellular vesicles. Samplers were centrifuged at 110,000× *g* for 90 min using an SW32Ti rotor in an Optima TMN-100XP ultracentrifuge. The pellets were resuspended in PBS and recentrifuged at 110,000× *g* for another 70 min with a Type100Ti fixed-angle rotor. All the mentioned centrifugations were performed at 4 °C. The final EV pellets were suspended in PBS and stored at −80 °C.

### 2.2. Western Blotting

The proteins of EVs in PBS buffer were isolated using RIPA lysis buffer (Beyotime, China), and supplemented with phenylmethylsulfonyl fluoride (PMSF) to a final concentration of 1 mM. The mixture was vibrated for several minutes and centrifuged at 12,000× *g* for 10 min. The protein concentration was quantitated using the BCA assay. The proteins CD63 and TSG101 were used to identify EVs, while calnexin (Canx, which only exists in cells) was used as a cell marker. The protein samples were mixed with 5× SDS loading buffer and boiled at 95 °C for 5 min. A total of 20 μg of each protein sample was loaded onto a 12% SDS-PAGE gel for electrophoresis at 120 V for 90 min. Proteins were transferred to polyvinylidene difluoride (PVDF) membranes (Life Technologies, Waltham, MA, USA) at a constant 260 mA for 2 h. The membranes were then blocked with 5% (*w/v*) skim milk powder in Tris-buffered saline with 0.05% (*v/v*) Tween-20 (TBST) at room temperature for 2 h. After blocking, the membranes were incubated at 4 °C overnight with primary antibodies against target proteins (anti-CD63,1:400, Cat No. 67605-1-Ig; anti-TSG101, 1:300, Cat No. 28283-1-AP; and anti-Canx 1:400, Cat No. 10427-2-AP). All primary antibodies were purchased from Proteintech (Wuhan, China) and diluted in TBST. After the primary antibodies were washed with TBST, the membranes were incubated with horseradish peroxidase-conjugated secondary anti-sheep/goat antibodies (diluted 1:2000) (Cat No. SA00001-4, Proteintech, Wuhan, China) at room temperature for 2 h. Finally, the immunoreactive membranes were washed several times with TBST and incubated with an enhanced chemiluminescence substrate (Millipore, Billerica, MA, USA). Immunoblots were captured using an imaging system.

### 2.3. Transmission Electron Microscopy (TEM) and Nanoparticle Tracking Analysis (NTA) 

To assess the morphology of EVs isolated from FF, EVs were resuspended in PBS buffer. The suspension was applied to a copper microgrid covered with a hydrophilic support film. EVs on a grid were stained with 3% phosphotungstic acid solution for 30 s and desiccated in air before observation. The shapes of EVs were captured and imaged using a transmission electron microscope (TECNAI G2 SPIRIT BIO, USA) at a voltage of 80 kV. For microparticle analysis, 10 μL of purified EVs were diluted in 1 mL PBS buffer and filtered through a 0.22 μm sterile filter. Each group of EVs was prepared in three replicates, and each sample was analyzed three times. The concentration and size distribution of EVs were determined using a NanoSight NS300 (Malvern Instruments, Malvern, UK), following manufacturer protocols.

### 2.4. The sRNA Extraction and sRNA Library Preparation

Six pools of EVs (three small and three large follicle pools) were collected and used. RNAiso for sRNA (TaKaRa, Beijing, China) was used to extract sRNA from EVs. Ultra-centrifuged EVs of FF were resuspended in RNAiso and the mixture was transferred to a new nuclease-free tube and vibrated thoroughly. The obtained RNAs were dissolved in nuclease-free water and stored at −80 °C. The total RNA quantity of samples was evaluated with an Agilent 2100 Bioanalyzer (Agilent Technologies, Santa Clara, CA, USA) and RNA concentration was measured using Qubit^®^ RNA Assay Kit in Qubit^®^ 2.0 Fluorometer (Life Technologies, Waltham, MA, USA). A total of 20 ng of small RNA per exosome sample was used for the sRNA sequencing library. Each sample library was constructed using the NEBNext^®^ Multiplex Small RNA Library Prep Set for Illumina^®^ (NEB, Ipswich, MA, USA) according to the manufacturer’s recommendations. In brief, based on the special structure of the -3′ and -5′ends of sRNAs, specific 3′ and 5′ adapters were directly ligated to the 3′ and 5’ends of sRNAs, respectively. First-strand cDNA was synthesized using RNA reverse transcriptase. PCR amplification was performed using the specific primers. DNA fragments in length of 140–160 bp were purified on an 8% PAGE gel to construct an sRNA library. Finally, the quality of the libraries was assessed using an Agilent Bioanalyzer 2100 system. Library construction and miRNA sequencing were performed by Novogene Company (Beijing, China).

### 2.5. Small RNA Sequencing and Date Processing 

Six small RNA libraries were sequenced on an Illumina Hiseq 2500 platform, and 50 bp single-end reads were generated. Reads containing ploy-N, with 5′ adapter contaminants, without 3′adapters or the insert tag, containing ploy A, T, G, or C, and low-quality reads were removed from raw data to obtain clean data. Subsequently, clean data were mapped to the reference goat genome sequence database [14] by Bowtie [15] using miRbase 20.0 as a reference. Known miRNAs were mapped to sRNA tags and modified using the software, mirdeep2. Custom scripts were used to obtain the miRNA counts and base bias on the first position of identified miRNAs of a certain length, and on each position of all identified miRNAs, respectively. To remove tags originating from protein-coding genes, repeat sequences, rRNAs, tRNAs, snRNAs, and snoRNAs, we mapped sRNA tags to the RepeatMasker or Rfam databases, or the data from cow and sheep species. To predict novel miRNAs, the characteristics of the hairpin structure of the miRNA precursor were used to explore the sRNA tags unannotated in the former steps and were integrated using the miREvo and mirdeep2 software sets. To ensure that each unique small RNA mapped to only one annotation, we followed the priority rule as follows: miRNA > rRNA > tRNA > snRNA > snoRNA > novel miRNA. The miRNA expression levels were estimated by transcript per million (TPM), as previously reported [16]:

Normalized expression = mapped read count/total reads × 1,000,000.(1)

Differential miRNA expression analysis of the two exosome groups was performed using the DESeq package in R. The adjusted *p-*value of < 0.01 and |log_2_(fold change, FC)| > 1 was set as the threshold for significantly differential expression by default.

### 2.6. Target Gene Prediction and Pathways Enrichment Analyses

The software mirDIP (http://ophid.utoronto.ca/mirDIP/, accessed on 5 November 2021) was used to predict the target genes of selected miRNA with high stringency [17]. The mirDIP software contains widely used target prediction programs, including DIANA, miRDB, TargetScan, and PicTar. To verify gene prediction, miRTarbase, an experimentally validated microRNA-target interaction database, was used to further ensure target genes were obtained. Gene Ontology (GO) enrichment analysis was performed to explore the functions of the predicted target gene candidates. GO terms with adjusted *p-*values *(Padj)* < 0.05, were considered significantly enriched target genes. The Kyoto Encyclopedia of Genes and Genomes (KEGG) pathways were used to annotate miRNA targets. The enrichment *p-*value was calculated using a hypergeometric distribution.

### 2.7. First Strand Synthesis of cDNA and Quantitative PCR (qPCR)

Total RNA was extracted using the RNAprep Pure Micro Kit (Tiangen, Beijing, China) and reverse transcription was performed using a RevertAid First Strand cDNA synthesis kit (Thermofisher, Waltham, MA, USA) according to the manufacturer’s instructions. For miRNA cDNA synthesis, stem-loop reverse primers for specific miRNAs were used. The q-PCR was performed using a 2 × RealStar Green Fast Mixture (Genstar, Shanghai, China) and reaction signals were detected using a Bio-Rad CFX96 q-PCR detection system (Bio-Rad, Hercules, CA, USA) with 96-well plates. The qPCR reaction conditions were 95 °C for 10 min, 95 °C for 10 s, 60 °C for 15 s, and 72 °C for 15 s, followed by 40 cycles of melting curve analysis to confirm the specificity of PCR amplification.

To validate the miRNA expression data obtained from small RNA sequencing, seven miRNAs (chi-miR-202-5p, chi-miR-424-5p, chi-miR-25-3p, chi-miR-22-3p, chi-miR-26a-5p, chi-miR-191-5p, and chi-miR-126-3p) were selected for expression analysis. The miRNA Ct value was normalized to the expression level of the spiked-in cel-miR-39 using the 2^−^^ΔΔCt^ method [18]. The stem-loop sequences and qPCR primer sequences of the miRNAs are listed in Table S2.

### 2.8. Statistical Analysis

All data are expressed as mean ± standard error of mean (SEM) from three independent replicates for each experiment. Data from the experiments were analyzed by *t*-test or one-way analysis of variance (ANOVA) followed by Duncan’s multiple range test comparisons using SPSS software (version 17.0; *p* < 0.05 or *p* < 0.01 indicate statistical significance.

## 3. Results

### 3.1. Characterization of EVs Isolated from FF

**Figure 1:**
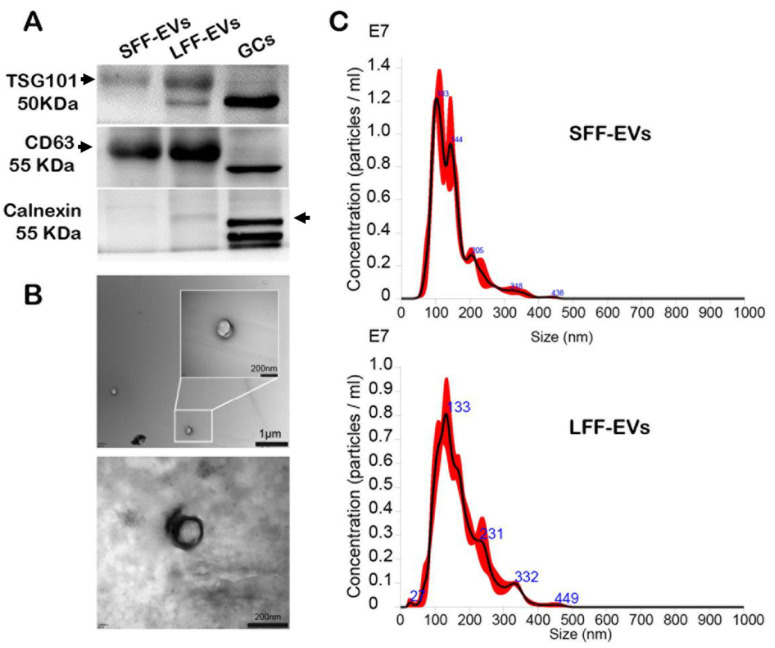
Identification and characterization of exosomes isolated from follicles. (**A**) Follicular fluid-derived extracellular vesicles (EVs) were identified by means of Western blotting; (**B**) the morphology of EVs were observed using transmission electron microscopy (TEM; Bar = 200 nm); (**C**) the size distribution of EVs was detected by Nanosight. SFF-EVs: small follicular fluid EVs; LFF-EVs: large follicular fluid EVs; GCs: granulosa cells.

The Western blot analysis revealed that EVs isolated from small and large goat FF were positive for two proteins (CD63 and TSG 101), which are well-known exosomal protein markers. Furthermore, the absence of calnexin showed the purity of EVs that were isolated using ultracentrifugation (Figure 1A). The pellet of EVs containing a bilayer membrane was observed by TEM (Figure 1B). Using nanoparticle tracking analysis (NTA), the size distribution of EVs showed that the diameter of EVs ranged from 30 to 200 nm. There were no distinguishable changes in the size distribution of EVs between the small and large follicles (Figure 1C).

### 3.2. The sRNA Profiles in EVs from FF of Small and Large Follicles

#### 3.2.1. The Sequence Length and Species Distributions of sRNA

**Figure 2:**
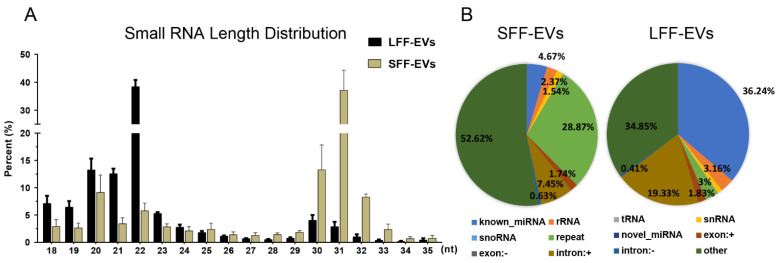
Overview of small RNA sequencing data. (**A**) Length distribution comparisons of clean reads between libraries, bars indicate the mean ± SEM of three independent libraries, nt: nucleotides. (**B**) Relative abundance of different sRNA species sequenced in SFF-EVs and LFF-EVs. SFF-EVs: small follicular fluid EVs; LFF-EVs: large follicular fluid EVs.

The length of sRNAs in EVs was less than 200 nucleotides (nt) (Figure S1). This indicated that large or long nucleotide sequences might be packaged with difficulty in EVs. Six sRNA libraries of EVs were subsequently sequenced using Illumina HiSeq2000, and a mean total of 14,432,117 and 13,985,482 raw reads of LFF-EVs and SFF-EVs were obtained, respectively (Table S1). After removing redundant structures and discarding sequences shorter than 17 nt or longer than 35 nt, a total of 13,735,702 and 12,824,584 clean reads in LFF-EVs and SFF-EVs, respectively, were determined for further analysis (Table S1). On average, in LFF-EV libraries, the length of sRNA was mostly distributed in the 20–22 nt range, while the common size of miRNAs was consistent at 22 nt in length (Figure 2A). However, most reads in SFF-EV libraries had a high percentage of approximately 31 nt, implying more than 70% non-miRNA components (Figure 2A). The sRNA annotation showed entirely different proportions of known miRNAs, rRNA, tRNAs, snRNAs, snoRNAs, novel miRNAs, exonic RNAs, and intronic RNAs between SFF-EVs and LFF-EVs (Figure 2B). A large proportion of repeats (28.87%) and unannotated sequences (other, 52.62%) significantly lowered the proportion of miRNA reads in SFF-EVs (miRNA, 4.67%) compared to LFF-EVs (miRNA 36.24%, repeat 3%, and other 34.85%). This indicated that various sRNAs were loaded into EVs at different follicular stages.

#### 3.2.2. The Expression Analysis of miRNAs in EVs

**Figure 3:**
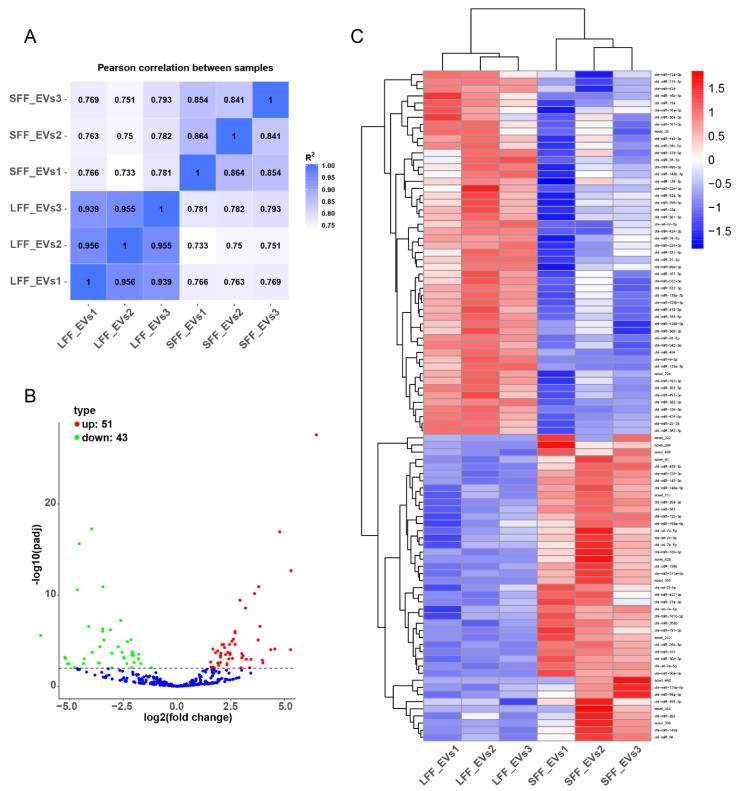
miRNAs expression analysis in EVs of follicular fluid (FF-EVs). (**A**) The Pearson correlation analysis between six libraries; (**B**) the volcano plot of differentially expressed miRNAs, with down-regulated miRNAs shown as green dots, whereas up-regulated miRNAs are shown as red dots; (**C**) the heatmap of all differentially expressed miRNAs.

All mapped clean reads were used for further classification and assessment. A total of 288 known and 54 predicted novel miRNAs and 189 known and 28 predicted novel miRNAs were identified in LFF-EVs and SFF-EVs, respectively. Similar expression patterns were observed between the LFF-EV and SFF-EV samples (Figure 3A). Among all miRNAs, a total of 94 miRNAs, 51 upregulated miRNAs, and 43 downregulated miRNAs were significantly differentially expressed in LFF-EVs compared to SFF-EVs (Figure 3B). The heatmap shows the results of unsupervised hierarchical clustering based on the significantly differentially expressed miRNAs (Figure 3C). 

### 3.3. The miRNA Profiles and Pathway Analysis of FF-EVs

**Figure 4:**
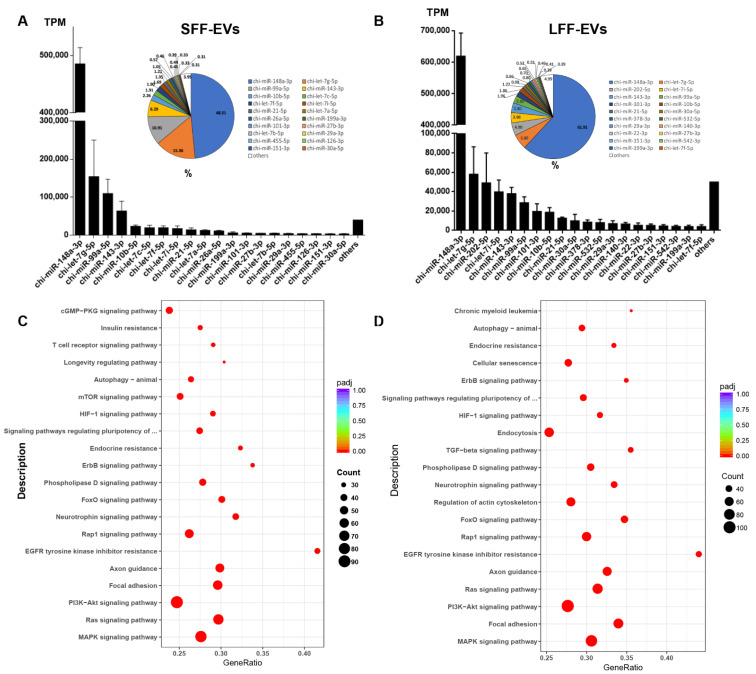
Expression distribution and predicted pathways of target genes of most enriched miRNAs. (**A**,**B**) The distribution of top 20 most expressed miRNAs in SFF-EVs and LFF-EVs; (**C**,**D**) the most relevant pathways regulated by the top 20 most expressed miRNAs identified in SFF-EVs and LFF-EVs, *padj*: corrected *p-*value.

In the present study, miRNAs in EVs were ranked according to total reads normalized to transcripts per million reads (TPM). We selected miRNAs with expression levels higher than 2000 read counts; in each group, the top 20 miRNAs accounted for more than 90% of all the EV-miRNAs (Figure 4A,B). The remaining hundreds of miRNAs were present at very low read counts and occupied only a very small percentage of total reads, indicating that these miRNAs with low reads might be less likely to have significant biological functions compared to other highly expressed miRNAs in EVs. Interestingly, chi-miR-148a-5p was the most abundant miRNA, which accounted for 61.91% and 48.51% of the miRNAs in LFF-EVs and SFF-EVs, respectively (Figure 4A,B). To investigate the biological functions of miRNAs that might be involved in different stages of follicular development by follicular fluid exosome miRNA, we analyzed the biological processes and molecular pathways of the most expressed miRNAs from LFF-EVs or SFF-EVs. In addition, we analyzed the putative target genes of the top 20 expressed miRNAs in the two groups and identified statistically over-represented pathways of these target genes. The significant enrichment pathways were selected using a corrected *p-*value *(padj*) < 0.05. The top 20 miRNAs were predicted to target 2814 and 2539 SFF-EV and LFF-EV genes, respectively (Figure 4C,D). Based on these predicted genes, similar KEGG pathway results were enriched in SFF-EVs and LFF-EVs. However, the insulin signaling pathway and progesterone-mediated oocyte maturation were specifically predicted in SFF-EVs. This implies that the EV-miRNAs play different roles during follicular development.

In order to determine gene functions in cellular and biological pathways that are potentially regulated by exosomal miRNAs in follicular development, we performed pathway analysis of predicted genes of miRNAs. Regardless of the low expression of differentially expressed miRNAs, we focused on the high expression of miRNAs in the two experimental groups. A total of seven upregulated (chi-miR-202-5p, chi-miR-22-3p, chi-miR-542-3p, chi-miR-532-5p, chi-miR-101-3p, chi-miR-30a-5p, and chi-miR-140-3p) and six downregulated (chi-let-7f-5p, chi-miR-99a-5p, chi-miR-455-5p, chi-miR-126-3p, chi-let-7a-5p, and chi-miR-26a-5p) miRNAs in LFF-EVs were determined (Table 1).

**Figure 5:**
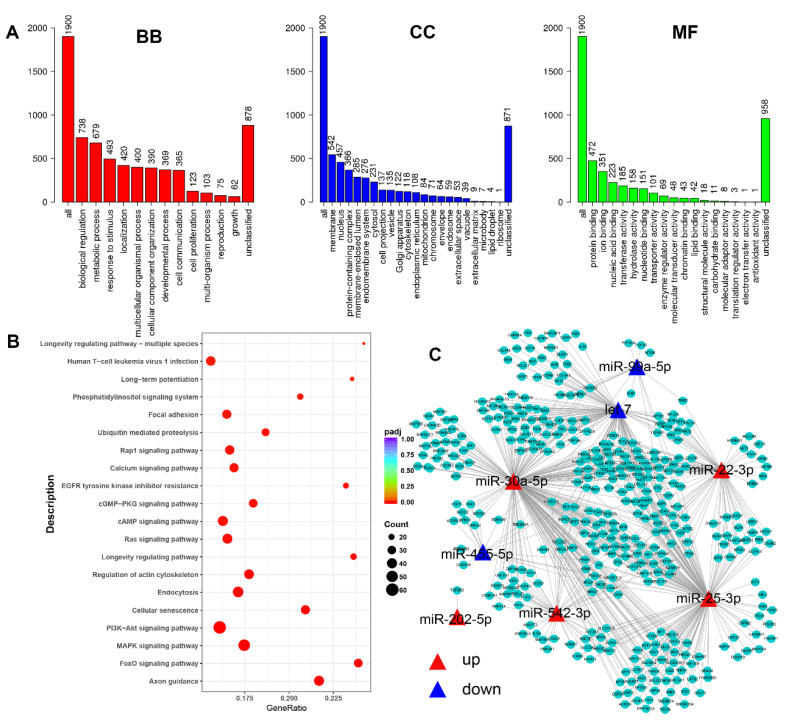
Bioinformatics analysis of differentially expressed miRNAs. (**A**) The Gene Ontology (GO) analysis of differentially expressed miRNAs predict target genes, three catalogues in different colors (biological process (BB), cellular component (CC) and molecular function (MF)); (**B**) the most enriched Kyoto Encyclopedia of Genes and Genomes (KEGG) pathways of predicted target genes, *padj*: adjust *p*-value; (**C**) the network of miRNA-genes of most related pathways.

For these 13 miRNAs, a total of 1948 target genes were predicted, and the most enriched terms were provided based on Gene Ontology (GO) analysis results. The 10 most enriched terms in every catalog (biological process, BB; cellular component, CC; and molecular function, MF) are shown in Figure 5A. Biological regulation, membranes, and protein binding were significantly enriched in the three catalogs (BB, CC, and MF). Additionally, the number of KEGG pathways was significantly enriched by the predicted genes. Here, we identified the top 20 pathways that were most significantly enriched. The targets were mainly involved in axon guidance, the FoxO signaling pathway, the MAPK signaling pathway, and the PI3K-AKT signaling pathway, inter alia (Figure 5B). The network of miRNAs and their target genes involved in the above pathways showed a complex relationship between miRNA and target genes (Figure 5C). Collectively, our results provide insight into the involvement of miRNAs in FF-EVs through several signaling pathways.

### 3.4. Validation of Sequencing Results Using RT-qPCR

**Figure 6:**
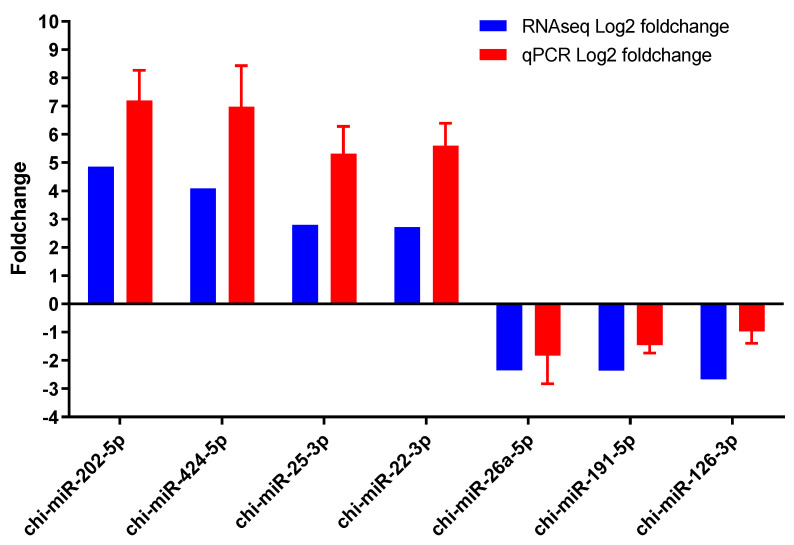
Validation of small RNA sequencing results by qPCR. Experiments were repeated three times. Bars indicate the mean ± SEM of three independent replicates.

The expression of seven upregulated and downregulated miRNAs was randomly selected to verify miRNA sequencing data. An accurate content of synthetic miRNA, cel-miR-39, as a normalized miRNA, was added to the total miRNA of exosomal LFF-EVs or SFF-EVs before first-strand cDNA synthesis. The results of the qPCR analysis show that the expression of these seven miRNAs was consistent with that obtained using RNA-seq (Figure 6). These data confirm that the sequencing data were reliable and accurate.

## 4. Discussion

Goat follicular growth is stimulated by gonadal hormones during the estrous cycle. Ovarian follicles provide a complex microenvironment for the interaction between somatic cells and oocytes. The follicular fluid composition shows dynamic changes during follicular growth and maturation. In the current study, we reported extremely different sRNA profiles in EVs between small and large follicles in goats, including sRNA sequence length distributions, relative abundance of different RNA species, and miRNA expression levels. To our knowledge, sRNA loading into EVs depends on the affinity of sRNA interacting with the EV membrane, which involves the sRNA sorting process [19]. This indicates a potentially unexplored change in EVs during follicular development.

The miRNA expression patterns were significantly different between SFF-EVs and LFF-EVs. Here, we accounted for the miRNA concentrations of EVs and showed that the 20 most enriched miRNAs in each group accounted for more than 90% of all miRNAs, since the remaining miRNAs formed only a small percentage of all miRNAs. The miRNAs are packaged into EVs, and it has been reported that low-abundance miRNAs (even less than one copy) load into a single EV [20]. It is difficult to accurately evaluate the function of the miRNAs with lower expression levels. Therefore, in this study, we focused on the different expression levels of the most enriched miRNAs in the two groups. 

The miRNAs profiles in follicular fluid EVs have been reported in different species by comparing the most enriched miRNAs in bovine [21], humans [22], horses [23], pigs [24] and goats. Some abundant miRNAs, such as miR-202-5p, miR-99a, miR-21-5p and miR-29a-5p, are shared in pigs and LFF-EVs of goats. The other species are different from each other. Different miRNAs profiles in EVs may show different mechanisms of actions in follicular cells in different species. In bovine, after incubation with follicular fluid, exosomes promoted cumulus expansion and oocytes maturation in vitro [10], while this was not observed in pigs [25].

The FoxO signaling pathway, MAPK signaling pathway, and PI3K-Akt signaling pathway were the most enriched by the target genes of 13 differentially expressed miRNAs. These signaling pathways play key roles in the proliferation and apoptosis of mammalian cells during follicular growth and in the promotion of primordial follicle activation [26,27]. The PI3K-Akt pathway is regulated by several miRNAs, such as miR-202-5p [28], miR-101 [29,30], and miR-22-3p [31], which are upregulated in LFF-EVs. It was reported that exosomes promote GCs’ proliferation through the P13K-AKT signaling [12] and MAPK signaling pathways [24]. In addition, miR-542-5p was previously found to be upregulated in GCs from bovine pre-ovulatory follicles compared to those in subordinate follicles [32]. Specifically, miR-202-5p is most significantly upregulated in LFF-EVs compared to SFF-EVs. In ruminants, miR-202-5p is highly expressed in large follicles in goats [33] and cattle [34]. In lower-order vertebrates, miR-202-5p knock-out causes the early steps of medaka oogenesis/folliculogenesis impairment and reduces the number of large follicles, leading to low fertility [35]. Moreover, miR-202-5p has been identified as a germline-specific miRNA [36,37,38] and can regulate ovarian hormone metabolism [39] and GC proliferation [40]. Thus, the biofunction of miR-202-5p in follicular development should be further explored. In mammalian ovaries, a large number of genes and related pathways participate in ovarian follicular growth until ovulation. Therefore, the molecular functions of predicted targets and pathways of miRNAs require further experimental verification.

In summary, we isolated and characterized EVs from the small and large follicles of goats. We revealed distinct EV-miRNA profiles from small to large follicles that are associated with follicular growth. Seven miRNAs with high levels were upregulated in large follicle EVs compared to small follicle EVs. Of these, miR-202-5p was shown as a follicle development potential marker packaged into EVs. Our study revealed that the EVs and their miRNA cargos changed dynamically over time, suggesting their potential use as biomarkers for cell-to-cell communication during follicular development. Finally, this study shows a specific FF-EV component and a set of miRNAs that may play key roles during follicle growth in goat ovaries.

## Figures and Tables

**Table 1:** The most significantly differently expressing miRNAs.

sRNA	log_2_ Fold Change (LFF-EVs/SFF-EVs)	*p-*Value	Adjusted *p-*Value	Regulation
chi-miR-202-5p	6.4674	8.44 × 10^−31^	2.85 × 10^−28^	Up
chi-miR-22-3p	3.5869	1.98 × 10^−12^	6.68 × 10^−11^	Up
chi-miR-542-3p	3.1663	9.48 × 10^−11^	2.67 × 10^−9^	Up
chi-miR-532-5p	2.1443	2.28 × 10^−6^	2.57 × 10^−5^	Up
chi-miR-101-3p	2.0369	5.97 × 10^−6^	1.41 × 10^−4^	Up
chi-miR-30a-5p	2.0034	6.47 × 10^−4^	5.55 × 10^−3^	Up
chi-miR-140-3p	1.3427	1.18 × 10^−5^	2.58 × 10^−4^	Up
chi-let-7f-5p	−1.7542	2.75 × 10^−3^	1.57 × 10^−2^	Down
chi-miR-99a-5p	−1.846	3.73 × 10^−4^	3.77 × 10^−3^	Down
chi-miR-455-5p	−2.1587	5.42 × 10^−7^	7.64 × 10^−6^	Down
chi-miR-126-3p	−3.4743	2.87 × 10^−13^	1.21 × 10^−11^	Down
chi-let-7a-5p	−3.4784	2.43 × 10^−8^	5.12 × 10^−7^	Down
chi-miR-26a-5p	−3.9945	3.28 × 10^−20^	5.54 × 10^−18^	Down

## Data Availability

Qiang Ding had full access to all the data in this study and takes responsibility for the integrity of the data and the accuracy of the data analysis. The data that support the findings of this study are available from the corresponding author upon reasonable request.

## Supplementary Materials

The following are available online at https://www.mdpi.com/article/10.3390/ani11113190/s1, Figure S1. Small or large follicles selected from goat ovaries; Figure S2. Length distribution of small RNAs in EVs; Table S1. Summary of sRNA data output and filter; Table S2. miRNA primer sequences used in this study.

## Abbreviations

BB	Biological Process
CC	Cellular Component
circRNA	circular RNA
COC	Cumulus-Oocyte Complex
EVs	Extracellular vesicles
FF	Follicular fluids
FoxO	Forkhead box O
GCs	Granulosa cells
GO	Gene Ontology
KEGG	Kyoto Encyclopedia of Genes and Genomes
lncRNA	long non-coding RNA
MAPK pathway	mitogen-activated protein kinase pathway
MF	Molecular Function
miRNA	microRNA
NTA	Nanoparticle Tracking Analysis
PI3K-AKT	phosphoinositide 3-kinase Active
piRNA	Piwi-interacting RNA
PMSF	Phenylmethylsulfonyl Fluoride
rRNA	Ribosomal RNA
snoRNA	small nucleolar RNA
snRNA	small nuclearRNA
TEM	Transmission electron microscopy
tRNA	Transfer RNA
BB	Biological Process
