# Supplementary material for: Comparison of MicroRNA Profiles in Extracellular Vesicles from Small and Large Goat Follicular Fluid
Source: Animals (Basel). 2021 Nov 8;11(11):3190. doi: 10.3390/ani11113190 (PMC8614480; doi:10.3390/ani11113190)
Supplement: Supplementary file 1 [file animals-11-03190-s001.zip › animals-1402121-supplementary.pdf]

Supplementary materials

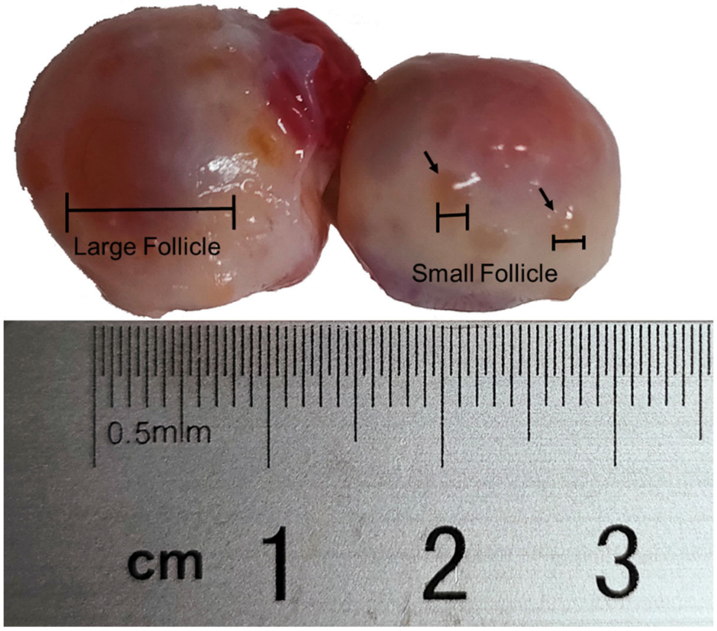

Figure S1. Small or large follicles selected from goat ovaries. Small follicles < 3 mm and large follicles > 5 mm in diameter.

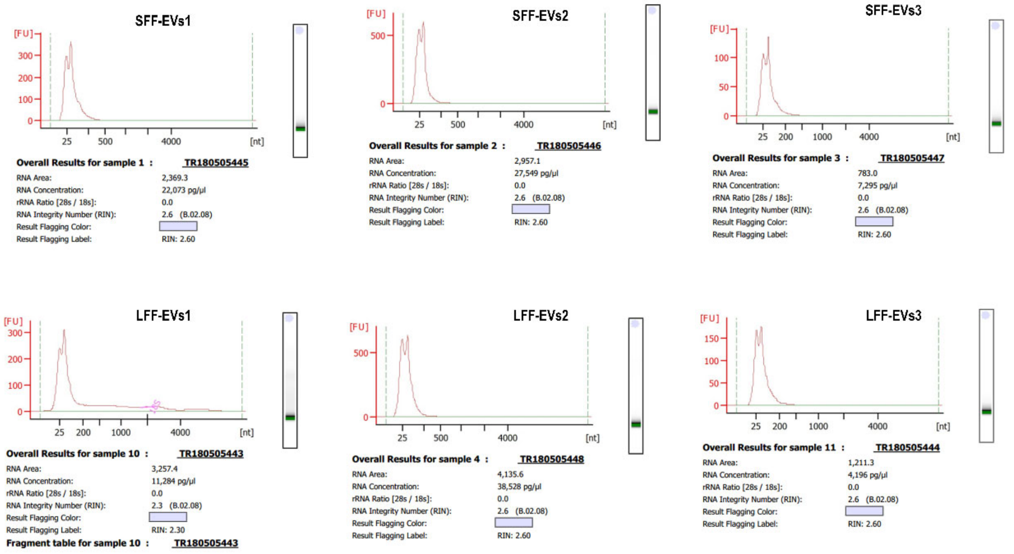

Figure S2 The length distribution and quality test of small RNAs in EVs

Table S1. The Summary of sRNA data output and filter

Sample	SFF			LFF		
	SFF-EVs1	SFF-EVs2	SFF-EVs3	LFF-EVs1	LFF-EVs2	LFF-EVs3
Raw Reads	13684470	14524981	13746996	15822734	13377814	14095802
Bases	0.684G	0.726G	0.687G	0.791G	0.669G	0.705G
Error rate	0.01%	0.01%	0.01%	0.01%	0.01%	0.01%
Q20	98.18%	98.08%	97.58%	98.15%	97.79%	98.07%
Q30	95.83%	95.61%	94.58%	95.83%	95.08%	95.60%
GC content	52.39%	53.09%	54.38%	51.61%	52.50%	51.96%
N% > 10%	96	91	99	98	101	106
low quality	207247 (1.51%)	236906 (1.63%)	231421 (1.68%)	233710 (1.48%)	168902 (1.26%)	224510 (1.59%)
5 adapter contamine	1885 (0.01%)	3700 (0.03%)	9424 (0.07%)	952 (0.01%)	1465 (0.01%)	1713 (0.01%)
3 adapter null or insert null	317022 (2.32%)	1261551 (8.69%)	1200198 (8.73%)	496030 (3.13%)	520645 (3.89%)	433209 (3.07%)
with ployA/T/G/C	1447 (0.01%)	4234 (0.03%)	7374 (0.05%)	2268 (0.01%)	3246 (0.02%)	2289 (0.02%)
clean reads	13156773 (96.14%)	13018499 (89.63%)	12298480 (89.46%)	15089676 (95.37%)	12683455 (94.81%)	13433975 (95.30%)

Table S2. The miRNA primers sequences were used in this study

	miRNAs	Primer sequences (5'-3')
Stem-loop RT primers	>chi-miR-202-5p	GTCGTATCCAGTGCAGGGTCCGAGGTATTCGCACTGGATACGACAAAGAA
	>chi-miR-424-5p	GTCGTATCCAGTGCAGGGTCCGAGGTATTCGCACTGGATACGACTCAAAA
	>chi-miR-25-3p	GTCGTATCCAGTGCAGGGTCCGAGGTATTCGCACTGGATACGACTCAGAC
	>chi-miR-22-3p	GTCGTATCCAGTGCAGGGTCCGAGGTATTCGCACTGGATACGACGTTCTT
	>chi-miR-126-3p	GTCGTATCCAGTGCAGGGTCCGAGGTATTCGCACTGGATACGACGCATTA
	>chi-miR-26a-5p	GTCGTATCCAGTGCAGGGTCCGAGGTATTCGCACTGGATACGACAGCCTA
	>chi-miR-191-5p	GTCGTATCCAGTGCAGGGTCCGAGGTATTCGCACTGGATACGACAGCTGC
	Cel-miRNA-39	GTCGTATCCAGTGCAGGGTCCGAGGTATTCGCACTGGATACGACCAAGCT
qPCR primers	qPCR>miR-202-5p	GCGGGCTTCCTATGCATATACT
	qPCR>miR-424-5p	GCGGGCCAGCAGCAATTCATGT
	qPCR>miR-25-3p	TGCCCGCATTGCACTTGTCTC
	qPCR>miR-22-3p	ACGGGCAAGCTGCCAGTTGAA
	qPCR>miR-126-3p	CGGGCTCGTACCGTGAGTAATA
	qPCR>miR-26a-5p	GCGGGCTTCAAGTAATCCAGGA
	qPCR>miR-191-5p	GGGCAACGGAATCCCAAAA
	Cel-miRNA-39	TCACCGGGTGTAAATCAGCT
	UR R	CAGTGCAGGGTCCGAGGT
